# Current Status and Future Perspectives of Betaine and Betaine-Based Natural Deep Eutectic Solvents: A Review

**DOI:** 10.3390/foods14234122

**Published:** 2025-12-01

**Authors:** Aylin Allahyari, Maryam Borji, Ali Jahanban-Esfahlan, Ali Khanalipour, Mahnaz Tabibiazar, Parisa Ahmadi

**Affiliations:** 1 Department of Food Science and Technology, Faculty of Nutrition and Food Science, Tabriz University of Medical Sciences, Tabriz 5165665811, Iran; ailinallahyarii79@gmail.com (A.A.); maryamborji031@gmail.com (M.B.); khanalipourali00@gmail.com (A.K.); 2 Student Research Committee, Tabriz University of Medical Sciences, Tabriz 5165665931, Iran; 3 Biotechnology Research Center, Tabriz University of Medical Sciences, Tabriz 5165665813, Iran; a.jahanban@gmail.com

**Keywords:** BET, food industry, natural deep eutectic solvents, pharmaceuticals

## Abstract

Betaine (BET)-based deep eutectic solvents (DESs) have emerged as promising substitutes for traditional organic solvents owing to their eco-friendly properties and versatility in various applications. This review provides a comprehensive overview of the current status and future perspectives of BET-based DESs, highlighting their definition, characteristics, and mechanisms of eutectic formation. The unique properties of BET, including its biodegradability and non-toxicity, make it an attractive hydrogen bond acceptor in the formulation of DESs. The review discusses common methods for preparing BET-based DESs and emphasizes their applications in extraction processes, catalysis, biocompatibility, and pharmaceutical applications. Additionally, challenges such as stability and fluidity limitations are addressed, along with regulatory and safety considerations. Future directions suggest an increasing industrial application of BET-based DESs in environmentally sustainable processes within the food and pharmaceutical sectors, underlining their potential as green solvents in next-generation chemical methodologies.

## 1. Introduction

Over the past two decades, the search for green solvents as sustainable alternatives to hazardous, toxic, and environmentally harmful solvents has become a central focus in green chemistry [1]. Deep eutectic solvents (DESs), first described by Abbott and colleagues in 2003 [2], have gained attention as “green solvents” due to their low volatility and reduced environmental impact compared to traditional organic solvents [3]. DESs are mixtures of two or more compounds that, when combined in specific ratios, display phase transition temperatures (melting points or glass transitions) markedly lower than those of their individual constituents [4]. These solvents have proven useful in a wide range of chemical processes, including extraction, biocatalysis, organic synthesis, electrochemical studies, CO_2_ capture, and materials research [4,5].

DESs are typically composed of hydrogen bond donors (HBDs) and hydrogen bond acceptors (HBAs). The HBDs often include plant-derived metabolites such as sugars, carboxylic acids, and amino acids, while HBAs are usually ammonium salts [6]. Choline chloride (ChCl) is a widely used HBA, but efforts to find cheaper and more accessible alternatives have intensified. Among these, betaine (BET), also known as *N*, *N*, *N*-trimethyl glycine or glycine BET (GB), has attracted significant interest. Initially isolated from sugar beets (*Beta vulgaris*) in the 19th century, BET is also found in wheat, wheat germ, spinach, microorganisms, and aquatic invertebrates. It is a zwitterionic molecule featuring a trimethylated quaternary ammonium group ((CH_3_)_3_N^+^) linked to an acetate group (CH_2_COO^−^), as shown in Table 1. The daily dietary intake of BET ranges from about 1 g to 2.5 g, with foods such as wheat and shellfish being particularly rich sources [7].

BET’s asymmetric structure, with a polar functional group, facilitates its strong eutectic interactions with various HBDs [6]. Recently, BET has been used as an HBA with compounds such as glycerol, 1,2-propanediol, lactic acid, levulinic acid, malic acid, citric acid, glucose, and sorbitol as HBDs in the formation of natural deep eutectic solvents (NADESs) [10]. NADES are a subclass of DES derived from naturally occurring metabolites, particularly those found in plants. They are typically composed of natural compounds such as sugars, amino acids, and organic acids, and polyols [11,12]. While both solvent types exhibit tunable physicochemical properties, NADES offer enhanced safety profiles, making them especially suitable for applications in pharmaceuticals, nutraceuticals, and food [12,13].

BET can be sustainably produced from sugar beet processing waste such as molasses and vinasse, promoting circular economy principles by valorizing agricultural by-products and enhancing the green credentials of BET-based DESs.

The multifunctionality of BET-based DESs has driven their application in diverse areas, including CO_2_ capture, enzyme stabilization, and metal production [14,15,16]. For example, ternary BET-based DESs demonstrate CO_2_ absorption capacities up to 0.207 g CO_2_/g DES and maintain high regenerability after repeated absorption–desorption cycles [14]. These solvents also improve enzyme thermostability, benefiting industrial bioprocesses [15] and have shown remarkable efficiency in protein extraction in aqueous two-phase systems, achieving up to 99.8% yield while minimizing reliance on costly reagents [17,18]. Additionally, BET DESs are used as both solvents and reactants in innovative slurry synthesis methods for cocrystal production, with tunable water content allowing control over molecular interactions to selectively produce binary or ternary cocrystals [19].

This study focuses on the value chain of BET as a low-cost agricultural waste for use as a green solvent. To identify relevant sources, databases and the Google Scholar search engine were used. Keywords related to “betaine”, “deep eutectic solvents”, extraction, and “pharmaceutical and food industries” were used in the search, which allowed for a more comprehensive analysis and broader coverage of the study. The aim is to explore the sources and extraction methods of BET from agrarian waste, focusing on its physicochemical and functional properties and associated health benefits. Furthermore, the formation of BET-based DES is considered, and the expanding role of BET-based DES, including their advantages and disadvantages compared to other eutectic solvents, is reviewed, with a focus on their applications in various sciences. By integrating waste valorization for BET recovery with the development of environmentally benign DESs, this work underscores a sustainable and health-oriented approach that bridges waste utilization, green chemistry, and functional food innovation.

## 2. Physicochemical Properties of BET

BET, with the chemical formula C_5_H_11_NO_2_ and a molecular weight of 117.15 g/mol, is a quaternary ammonium compound also known as GB, lycine, and oxyneurine [20]. It exists as a zwitterion, having a positively charged nitrogen atom (N^+^) adjacent to a negatively charged carboxylate group (COO^−^). BET has been characterized as a methylamine due to its three chemically reactive methyl groups [21].

BET appears as a white crystalline or granular powder that is non-volatile, odorless, and has a slightly sweet taste. It is highly soluble in water and alcohols and is hygroscopic, meaning it readily absorbs moisture from the air. BET is stable under normal conditions, resistant to oxidation, but sensitive to strong oxidizing agents. Its melting point is around 301–305 °C, where it decomposes. The glass transition temperature (Tg) of pure BET is not commonly specified because it is typically found as a crystalline solid or dissolved in aqueous solutions [22]. However, when BET is part of composite materials or DES, the Tg can be characterized by differential scanning calorimetry. BET-based DES exhibits very low glass transition temperatures and behaves as a supercooled liquid with unique thermal properties [23]. In pharmaceutical and food science contexts, Tg is most relevant when BET is combined in formulations or amorphous states, where it depends on moisture content, formulation, and physical state. In the United States, BET is generally recognized as safe (GRAS), whereas in Europe, the European Commission has authorized its application in food at a minimum amount of 500 mg per serving. Commercially, BET exists in three forms: natural anhydrous BET, synthetic anhydrous BET, and BET hydrochloride [21]. The main properties of BET are presented in Table 1.

Various BET analogues are found naturally in plants and marine organisms, often playing key roles in stress adaptation and osmotic regulation. Proline BET (stachydrine) is abundant (up to 100 μmol/g dry mass) in some citrus varieties, increasing up to threefold under salt stress [24]. Trigonelline is present in coffee beans, tomatoes, and alfalfa, with levels also rising in stressed plants [25,26,27]. Sulfonium BET analogues like di-methyl sulfoniopropionate (DMSP) and the arsenic-containing arseno-BET occur in marine bacteria and phytoplankton and accumulate in marine animals [28,29]. These analogues—proline BET, trigonelline, and arseno-BET—are detectable in human plasma and urine primarily from dietary intake and are readily absorbed but differ from BET by not accumulating in the kidney [30,31,32]. BET is found on the market in the chemical forms of BET anhydrous, BET monohydrate, BET hydrochloride, and cocamidopropyl BET. The first two are widely used in the food, feed, and pharmaceutical sectors. BET hydrochloride, which is its acid salt form, is mainly used in dietary supplements and medical applications due to its role as a methyl donor and osmolyte. Cocamidopropyl BET, a surfactant-derived form, is widely used in shampoos and body washes due to its foaming and conditioning properties.

## 3. Healthy and Functional Properties of BET

BET is known as a natural and essential methyl donor that maintains methionine homeostasis in cells. It is formed from the oxidation of choline in cells and can also be obtained from dietary sources [8]. BET can be irreversibly produced in the human body from free choline through the action of choline dehydrogenase enzyme. This process is catalyzed by a series of enzymatic reactions that occur primarily in the mitochondria of liver and kidney cells [33]. Several studies have demonstrated the metabolic and functional benefits of BET over ChCl. M. Hruby et al. (2005) emphasized that BET is a more direct methyl group donor, as choline must first be converted to BET, making it more metabolically efficient [34].

However, endogenous production of BET is usually inadequate to satisfy daily requirements; thus, dietary BET intake is deemed necessary [21,35]. Humans obtain BET either directly from foods containing BET or from choline-containing compounds [36]. BET content in foods varies and is generally related to growing conditions and osmotic stress. BET helps maintain intracellular osmotic pressure like other electrolytes. It does not bind significantly to protein surfaces, allowing it to control surface tension effectively [33]. Recent studies have identified BET as a potential therapeutic agent for alcohol-induced and metabolic-associated liver diseases owing to its low cost, high tolerability, and efficacy [9,37]. BET protects against alcohol-induced liver damage, including hepatic steatosis (fatty liver), apoptosis (cell death), and protein damage. It also helps prevent and attenuate metabolic-associated fatty liver disease by maintaining methionine metabolism, lowering homocysteine, and supporting methylation balance in liver cells. Additionally, BET preserves gut integrity and adipose tissue function, both of which are important for liver health [37]. In alcohol-fed mice, BET decreases hyperhomocysteinemia, endoplasmic reticulum stress, and liver injury [38]. Elevated plasma homocysteine is a risk factor for cardiovascular disease and stroke. BET lowers plasma homocysteine levels by donating methyl groups to convert homocysteine back to methionine via the enzyme BET-homocysteine methyltransferase (BHMT), which metabolizes up to 50% of homocysteine in the liver, kidneys, white adipose tissue, and intestine [7]. Together with the vitamin B_12_ and folate-dependent methionine synthase pathway, BET supplementation may help reduce cardiovascular disease risk [39].

BET also improves insulin sensitivity and glucose metabolism. It reduces fat accumulation inside muscle cells and lowers abdominal fat, aiding lipid metabolism by modulating cholesterol and triglyceride levels [40]. The Gao, Zhang [41] meta-analysis supports that BET supplementation can reduce body fat mass and fat percentage without affecting overall weight or BMI, but subsequent reviews highlight controversial or inconsistent evidence requiring more research [42]. Moreover, animal studies have shown positive effects of BET on lean mass and fat reduction; similar benefits have not been consistently observed in humans [40].

BET exhibits unique antioxidant properties distinct from traditional antioxidants because it has little free radical scavenging activity itself [43]. Instead, its antioxidant effects are primarily indirect: BET enhances the body’s nonenzymatic antioxidant defenses by regulating the methionine–homocysteine cycle, increasing levels of compounds like S-adenosylmethionine (SAM) and methionine, which contribute to cellular antioxidant capacity. The three methyl groups in BET are crucial, as they enable it to form a protective hydrophobic and hydrophilic membrane around cells, helping to prevent excessive reactive oxygen species (ROS) generation and cell damage. BET treatment increases the function of major antioxidant enzymes, including glutathione peroxidase (GPx), superoxide dismutase (SOD), and catalase (CAT), enhancing cellular defense against oxidative injury. In animal studies, BET has protected brain, liver, and kidney tissues against oxidative stress-induced damage, often outperforming or complementing traditional antioxidants. Compared to other osmoprotectants like proline, BET’s antioxidant effect is less direct but effective through metabolic and membrane-protective mechanisms. BET supplementation is widely applied in animal nutrition to improve production performance, nutrient utilization, product quality, resilience during heat and osmotic stress, and overall well-being in livestock [44]. BET also supports brain health by restoring methylation potential, preventing axonal damage, and possibly reducing psychiatric symptoms such as anhedonia (lack of pleasure) [45]. It supports epigenetic regulation and activates neuroprotective gene expression. Additionally, BET improves myocardial (heart muscle) function, prevents damage from toxins, and enhances muscle strength and endurance, improving athletic performance via benefits to both anaerobic and aerobic metabolism. BET supplementation reduces the harmful effects of heat stress, supports fetal development during pregnancy by normalizing growth and reducing fat deposition, and exerts protective effects on the pancreas and kidneys [9,46].

In food matrices, while phenolic antioxidants often show high activity in chemical assays (like DPPH or ABTS radical scavenging), BET’s antioxidant effects are more physiological and stabilizing rather than direct radical quenching. For example, BET has been demonstrated to maintain firmness, minimize weight loss, and preserve antioxidant enzyme activities in stored fruits, providing protective benefits complementary to classical antioxidants. BET boosts the activity of major antioxidant enzymes, including SOD, CAT, and GPx, which work collectively to reduce oxidative damage in cells. Numerous reports demonstrate the effectiveness of BET coatings in increasing the shelf life and post-harvest quality of fruits such as strawberries due to the stabilization of cell membranes and protection of proteins against oxidative stress. This results in the maintenance of firmness in fruit and vegetable tissue and reduction in chilling injury [47,48]. BET or GB has been extensively studied for use in fruit storage as an important osmotic regulator and compatible solute. Its role in reducing fruit softening after harvest is well proven [49]. Proline and GB are essential metabolites that enhance plant tolerance to multiple abiotic stresses through osmotic adjustment, membrane and protein stabilization, antioxidant defense activation, and molecular chaperoning. They help activate antioxidant defense systems by upregulating enzymes such as SOD, CAT, and GPX, which reduce oxidative damage caused by stress-induced ROS [50]. A previous report showed that adding BET (1 g/kg) to a methionine-deficient diet significantly improved antioxidant defenses and meat quality by decreasing lipid peroxidation in the breast muscles of broiler chickens [51]. BET treatment at 20 mM concentration has been shown to reduce the accumulation of harmful ROS such as hydrogen peroxide (H_2_O_2_) by enhancing antioxidant enzyme activities, including CAT, ascorbate peroxidase (APX), and peroxidase (POD). These enzymes convert H_2_O_2_ into water and oxygen, thereby reducing oxidative stress. GB accumulates in chloroplasts, mitochondria, and the cytosol, stabilizing photosynthetic machinery and protecting membranes and proteins from ROS-induced oxidative damage [52].

As fruits mature, phenolic and flavonoid contents increase significantly. For example, in strawberries, total phenolics rise from about 491 mg to 1884 mg gallic acid equivalents (GAE) per 100 g dry weight (DW), and the total flavonoid content rises from 83 mg to 327 mg catechin equivalents (CE) per 100 g DW from unripe to fully ripe fruit. BET treatments in postharvest fruits like blueberries and pistachios have been shown to inhibit the degradation of phenolic and flavonoid compounds, preserving antioxidant components during storage. By preserving these compounds, BET helps sustain antioxidant defenses, color, and nutritional value in fruits during storage and handling. BET enhances food shelf life by protecting against oxidative stress, inhibiting enzymatic browning, maintaining firmness, and preserving nutritional and sensory quality, making it a promising natural preservative and postharvest treatment [53].

BET can be used in various ways to enhance plant growth, stress tolerance, and food preservation. Foliar application, which involves spraying BET solution directly onto leaves, is the most common method, typically at concentrations ranging from 0.5% to 2% (5000 to 20,000 ppm). Timing is critical and often coincides with key growth stages such as flowering or early fruit development. Foliar application improves plant water retention, antioxidant activity, and overall resilience to abiotic stresses like drought and salinity. BET can also be applied to soil via irrigation water or direct incorporation. In controlled environments such as hydroponics, BET added to nutrient solutions ensures steady root uptake, enhancing growth and nutrient content, as shown in lettuce studies. BET sprays on harvested fruits and vegetables reduce chilling injury, slow browning, and extend shelf life [54,55,56,57]. Soaking seeds in BET solution before planting can improve germination rates, seedling vigor, and stress tolerance [57]. These methods help plants better withstand abiotic stresses, maintain quality, and increase productivity, making BET a versatile and valuable bioactive compound for sustainable crop management.

Additionally, BET plays an effective role in reducing heavy metal stress, mitigating the harmful effects of heavy metals on plants [58]. BET contributes to osmotic defense, particularly in the kidneys, liver, and brain, accumulating in large amounts within cells without impairing cellular activity. This osmoprotective role helps preserve cells, proteins, and enzymes under osmotic stress [59]. For example, spinach grown in saltwater can accumulate BET up to 3% of its fresh weight, supporting chloroplast photosynthesis under high salinity [60]. In aquaculture, BET is incorporated into aquaculture feed as an osmoregulator to protect fish transitioning through different salinity waters [21]. Furthermore, BET is employed in certain industrial processes as a stabilizing, emulsifying, and surfactant agent [61,62]. Common foods rich in BET and their approximate BET content per 100 g are presented in Table 2. Depending on cooking methods, for instance, boiling causes the greatest loss of BET [63]. When incorporated as a food additive, BET shows good bioavailability and is metabolized within the mitochondria of liver and kidney cells, first to dimethylglycine and then further to sarcosine [33,64].

Overall, in food matrices, BET contributes to oxidative stability by supporting enzymatic antioxidant defenses and maintaining structural integrity, which differs from common antioxidants that primarily act by direct free radical neutralization. This makes BET a valuable natural preservative and functional ingredient for enhancing food quality and shelf life.

Based on previous studies, BET has exhibited notable antimicrobial properties that make it valuable for food preservation and safety. Amphiphilic BET esters are quaternary ammonium compounds (QACs) with a rapid microbicidal effect, which spontaneously hydrolyze into non-toxic products, thus being referred to as soft antimicrobial agents [65]. BET-based compounds, such as polycaprolactam-BET and polyhexamide-BET, demonstrate bacteriostatic and fungistatic effects against a wide range of bacteria, including *Staphylococcus aureus*, *Escherichia coli*, *Enterococcus faecalis*, *Klebsiella pneumoniae*, and *Pseudomonas aeruginosa*, as well as fungi [66].

Minimum inhibitory concentrations for these BET derivatives range from 0.5 to 8 mg/L for bacteria, and they also significantly inhibit biofilm formation [67]. BET esters, quaternary ammonium compounds derived from BET, exhibit robust microbicidal activity comparable to conventional disinfectants but hydrolyze into non-toxic metabolites, enhancing their safety for food and body surface applications [68]. The antimicrobial efficacy of BET is related to its molecular structure, specifically the length of the alkyl chain in its derivatives. For example, BET derivatives with shorter tails should be used for disinfection in the presence of proteins at lower temperatures [65]. BET has been successfully incorporated into edible films and coatings (e.g., BET-gelatin films) to prevent microbial spoilage of foods like fish and fruits, slowing fungal and bacterial growth during storage.

Overall, BET and its derivatives offer effective antimicrobial action by disrupting microbial cell membranes, inhibiting growth and biofilm formation, thereby enhancing food safety while being non-toxic and compatible with food systems [69].

BET also exhibits antiviral effects through several mechanisms, particularly demonstrated in studies on hepatitis B virus (HBV) [70]. It reduces the release of HBV DNA and viral antigens by suppressing the expression of GRP78, a protein that facilitates viral replication. Moreover, it may enhance the antiviral effects of interferon alpha by boosting the production of antiviral proteins. Beyond HBV, BET’s anti-inflammatory and antioxidant properties help reduce tissue damage caused by viral infections by suppressing pro-inflammatory cytokines and oxidative stress. Its ability to inhibit nuclear factor-κB (NF-κB) activity and downstream inflammatory genes contributes to both its antiviral and anti-inflammatory actions [70,71,72]. In a study conducted by Blinov et al., selenium nanoparticles (Se NPs) stabilized with cocamidopropyl betaine were synthesized as a surfactant. The synthesized cocamidopropyl BET–BET-stabilized Se NPs showed an excellent stability in a real system (liquid soap) [73].

A zwitterionic BET-based amphiphilic polymer as a stable self-assembled network in aqueous solutions was synthesized. The network structure of the polymer maintains and strengthens even in the presence of salt by balancing positive and negative charges. These properties make BET-based polymers promising candidates for industrial applications, especially in enhanced oil recovery processes under high salinity conditions [74].

Some of BET’s functional applications are summarized in Table 3.

## 4. BET Production/Extraction

Natural extraction of BET is important to meet market demands for high-quality, sustainable, and bioactive ingredients, supporting a growing global market projected to exceed $5 billion by 2027. BET can be extracted from various natural resources, including sugar beet molasses (3–8% content), fruit peels, and whole grains. This diversity ensures a consistent supply and reduces reliance on a single source. Molasses, the primary and most valuable by-product of beet sugar refining, has long served as the main natural source of BET. Vinasse, a by-product produced during the ethanol fermentation of molasses, also represents an important source [88]. BET is not destroyed or lost during the processing of crystalline sugar from sugar beet and accumulates in molasses almost without loss [89,90]. Generally, BET levels in sugar beet peak during autumn months, and drought conditions also increase BET content. Below are briefly reviewed BET extraction methods:

### 4.1. Reactive Extraction Using Dinonylnaphthalene Disulfonic Acid (DNNDSA)

DNNDSA dissolved in an organic solvent, such as toluene, is used to extract BET from aqueous solutions of sugar beet byproducts. Extraction efficiencies of around 67–71% in one step have been achieved. The extracted BET can be stripped from the organic phase using sodium hydroxide, recovering about 47–54% of the initial BET in a single extraction and stripping step. This method is noted for its high efficiency, ease of use, minimal energy requirements, and economical operation. Extraction performance improves with higher extractant concentration and slightly with increased temperature, while the polarity of the organic solvent also affects efficiency [88,91].

A previous study tested three pretreatments before separation: (I) no pretreatment, (II) acidification (pH adjustment), and (III) alcohol fermentation. The highest increase in BET content was achieved without pretreatment, raising BET from 62.76% dry matter (DM) in the starting sample to 85.16% DM in the final product. Acidification resulted in a lower increase (67.68% DM), and alcohol fermentation did not significantly change BET content. This suggests omitting pretreatment can yield higher purity BET during separation [91].

### 4.2. Alcohol Extraction

Wastewater from sugar beet processing is concentrated and extracted with alcohol solvents, commonly ethyl alcohol. The alcoholic extract, which can contain 40–45% BET on a dry matter basis, is treated with acid (usually hydrochloric acid) to form BET acid salts that crystallize out. The solution is passed through hydrogen ion exchange resins to enrich the BET concentration before acid treatment. Purification steps include activated carbon treatment and anion exchange to remove impurities. The resulting BET is then refined by crystallization to produce a high-purity product. This method reduces acid use and avoids some complications from organic impurities but requires acid-resistant equipment [92].

### 4.3. Chromatographic Separation and Crystallization

Diluted beet molasses or vinasse with intermediate solids content undergoes chromatographic separation to isolate and concentrate the BET-rich fraction, often involving multiple recirculations for improved yield. Crystallization is then used to recover the BET hydrate. Overall, BET recovery can exceed 80% with this method [93]. A widely used industrial process is the molasses desugaring process [94], which employs a chromatographic column filled with a cation exchange resin (e.g., polystyrene sulfonate crosslinked with divinylbenzene). Molasses is diluted to 25–50% solids and passed through the column, where water elutes BET from sugars and other components. Successive feeds allow partial overlap, and BET-rich fractions are collected continuously. Waste and sugar fractions are recycled or discarded accordingly. The eluted BET fraction is concentrated by evaporation and crystallized as monohydrate or anhydrous BET. This process achieves high yield (up to 80%) and purity (~99.8%) suitable for commercial use [94].

### 4.4. Membrane Technology

This technique enables separation without mixing phases, reducing emulsification and simplifying the process. The membrane contactor uses hollow fibers that provide a large surface area for controlled mass transfer between two immiscible phases—usually an aqueous phase containing BET and an organic solvent phase that selectively extracts BET. Extraction uses an organic phase with extractants like dinonylnaphthalene sulfonic acid (DNNSA) dissolved in solvents such as n-heptane, alongside a stripping phase (e.g., sodium hydroxide solution) to recover BET. Operating parameters—flow rate, pH, temperature, and solvent choice—are optimized to maximize efficiency and purity. Membrane contactors offer advantages over traditional chromatographic or evaporative methods by lowering energy use, improving selectivity, enabling continuous processing, and efficiently treating complex molasses mixtures. Hollow fibers allow phase contact without mixing, and BET selectively transfers from aqueous to organic phase through the membrane interface [95,96]. Table 4 provides a comparative analysis of extraction methods.

## 5. Fundamentals of BET-Based Natural Deep Eutectic Solvents

### 5.1. BET-Based DESs

The most important HBAs in the formation of DES are typically quaternary ammonium salts and other compounds with positively charged or electron-rich centers that can accept hydrogen bonds from HBDs. ChCl, BET, and various ammonium or phosphonium salts such as tetra-alkyl ammonium and tetra-alkyl phosphonium chlorides or bromides, primarily characterized by their ability to structurally accept hydrogen bonds and stabilize the eutectic mixture via strong intermolecular interactions [97,98,99].

BET is an important component of various DES and NADES mixtures, but the mechanism of its formation is of interest. BET is a zwitterion, i.e., it possesses both formal positive and negative charges. As such, BET should establish strong ion–ion interactions with itself. Because ion–ion interactions are much stronger than hydrogen bonding, this assumption contradicts the formation of DES from BET, which is based on hydrogen bonding. But in practice, due to the asymmetric structure of BET, the positive charge of BET is shielded by methyl groups, which may inhibit electrostatic interactions with itself. Therefore, this compound becomes a strong hydrogen bond acceptor due to the polarity imbalance and the presence of the carboxylate group [100,101].

Generally, DESs and NADESs are prepared by weighing the specific molar ratios of HBDs and HBAs and heating at temperatures ranging from 50 °C to 85 °C until the final homogeneous liquid formation. Alternative preparation methods include exposure to irradiation (microwave and ultrasound) and mechanical methods (grinding), or a combination of temperature and mechanical forces (twin screw extrusion) [102,103,104,105]. After mixing, components may be ground with a mortar and pestle at room temperature until homogeneity is achieved [106].

Based on previous studies, the physicochemical properties of DES, including viscosity, density, melting point, electrical conductivity, thermal stability, polarity, hydrogen bond strength, and water content, determine its applications in various fields [107].

The strength and stability of DES depend heavily on the interaction between the HBA and HBD, with the HBA’s structure—like the size of its central atom, the length of alkyl chains, and the nature of its anion—playing a key role in hydrogen bonding and solvent properties [108].

BET-based DES generally have higher viscosity, density, and surface tension due to stronger hydrogen bonding and molecular interactions compared to ChCl-based DES [109].

In terms of electrical conductivity, when BET is combined with an acid such as lactic acid, the interactions can lead to the production of additional ions in solution, thereby increasing electrical conductivity. The hydrogen bonding between BET and lactic acid is less extensive than that in BET-glycerin or BET-xylitol systems, allowing for a higher concentration of free ions. Additionally, the introduction of up to 50% water into these DES systems significantly increases their electrical conductivity [110]. However, high density and viscosity frequently decrease fluidity and complicate mass transfer in continuous operations and dissolution processes [91,97,98].

Both types of DES show significant melting point depression compared to their individual components. The eutectic temperatures are often similar, but BET-based DES can maintain a liquid state and stability over a broader temperature range.

Water influences both systems but differently; it can weaken interactions in ChCl-based DES while it enhances interactions in betaine-based DES near eutectic compositions, affecting viscosity and extraction efficiency.

BET-based DESs can be easily prepared with high purity and without by-product formation [111,112]. Its DESs exhibit tunable properties and are efficient media for protein extraction and metal recovery [16,113], biocatalysis of antimicrobial polymers without toxic [100,114], and enzymatic degradation of pollutants such as synthetic dyes and phenolic compounds in wastewater [14]. They also show potential in pharmaceuticals by improving drug solubility and bioavailability, and in agriculture for green isolation of bioactive compounds [113,115]. BET-based DESs have been used as extraction media for phenolic compounds from spent coffee grounds (SCG), a food waste product. Moreover, they enable the selective removal of undesired molecules such as heavy metals and pesticides from food, enhancing safety and quality. BET-DESs are being explored for biodegradable food packaging materials, offering eco-friendly alternatives to plastics while maintaining food quality [105]. In aqueous two-phase systems (ATPS), BET-urea DES enables efficient extraction of Bovine Serum Albumin (BSA) protein with 99.82% efficiency and minimal protein conformational changes. Back extraction and stability studies confirm their practical value for biotechnological applications [116]. Varying BET-to-HBD ratios (e.g., 1:3.5–1:6 for BET-1,2-propanediol DES) adjusts viscosity, density, and surface tension [117]. Water content modulates molecular interactions and hydration, while temperature adjustments optimize hydrogen bonding networks and fluidity, balancing solvent stability and reactivity [118]. Quantum chemistry and molecular dynamics simulations provide insights into DES molecular structure, interaction energies, and dynamics, allowing predictive design to tailor DESs for specific industrial needs such as enhanced extraction efficiency, thermal stability, or reduced viscosity [19,118]. Challenges such as long-term stability need to be addressed to scale BET-based DES industrially [16]. With further research, BET-DESs could revolutionize sustainable practices across multiple sectors [14,100].

### 5.2. Characterization Techniques for BET-Based DES

Characterization techniques for BET-based DES focus on understanding their thermophysical properties, molecular interactions, and structural features, which are crucial for various applications. Fourier-transform infrared spectroscopy (FTIR) and nuclear magnetic resonance (NMR) spectroscopy are extensively used to confirm hydrogen bond formation and molecular structure. FTIR identifies hydrogen bonding patterns and functional groups within the DES, such as the interaction of CO_2_ with –NH_2_/–OH groups during CO_2_ capture [107]. NMR provides insights into the local chemical environment of atoms, helping to elucidate the interactions between BET and its HBD counterparts [108].

Thermal stability and melting point of DES are primarily determined using the thermogravimetric analysis (TGA) and differential scanning calorimetry (DSC) analysis.

TGA measures the weight loss of the DES sample as the temperature increases. The decomposition temperature (T_dcp_), where the sample loses 10% of its original weight, indicates thermal stability. TGA can be performed dynamically with increasing temperature or isothermally at a constant temperature over time to assess long-term stability. DSC measures heat flow associated with phase transitions such as melting. It detects melting points and crystallization temperatures of DES to determine their phase behavior. DSC profiles show endothermic peaks corresponding to melting. Together, TGA provides data on thermal degradation limits, while DSC reveals melting and crystallization temperatures [119,120].

Viscosity and flow behavior are assessed using viscometers or rheometers to characterize the fluid dynamics critical for processing and application. These studies reveal how the flow regime changes with different HBD combinations, indicating the influence of molecular structure on the solvent’s behavior under stress [107]. Viscosity affects mass transfer, while polarity affects the solvation power.

Studies have shown that the density of these solvents generally varies between 1.2 and 1.3 g cm^−3^ at 20 °C, while dynamic viscosities can vary significantly depending on the composition, with some mixtures reaching up to 600 mPa·s at elevated temperatures [114,121].

Electrical conductivity and pH measurements reveal ionization and dissociation behavior essential for applications involving catalysis or electrochemistry.

Gravimetric or Karl Fischer titration methods assess water content, while water activity measurements help understand solvent interactions and biocompatibility, especially important for enzymatic applications.

Computational methods, particularly molecular simulations and machine learning, have played a pivotal role in DES research over the past five years. These approaches complement experimental data by predicting key properties such as density, phase behavior, and hydrogen bonding. By leveraging computational modeling, researchers have resolved several fundamental questions about the relationship between DES structure and their physicochemical properties, providing guidance for the rational design of tailor-made deep eutectic solvents and greatly accelerating the development of new formulations [122].

Together, these characterization techniques provide a comprehensive understanding of BET-based DES, guiding their design, optimization, and application in various fields.

## 6. Current Applications of BET-Based DES

The diverse application potential of BET-based DESs has been reported [123,124,125,126,127,128], and some studies on their properties are also available [129,130,131]. As discussed in the following subsections, the growing body of research suggests a bright future for BET-based DESs in various fields.

### 6.1. Extraction/Separation of Compounds

BET-based DESs exhibit unusual properties when mixed with various organic substances, enhancing their potential for diverse applications [97,100].

The polarity and preparation methods of BET-based DESs have been extensively studied to optimize their use in separations and extractions of different components [3].

**Figure 1:**
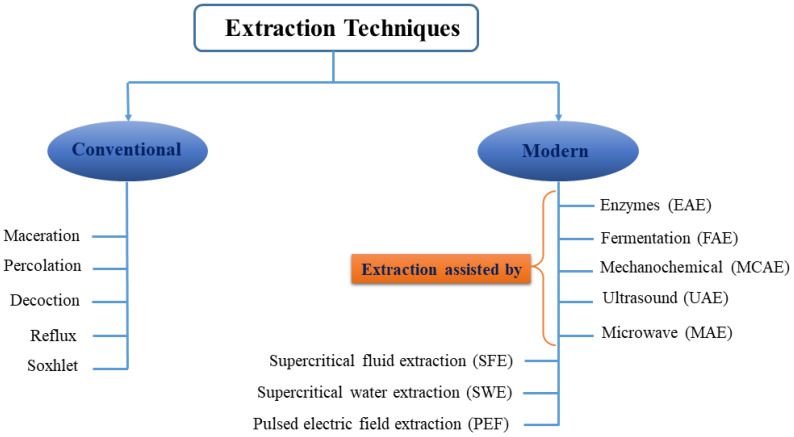
Techniques Normally Used for the Extraction of Bioactive Compounds.

Isolation of compounds possessing bioactive potential, especially from agricultural wastes, has received much attention for creating a sustainable and value-added food system. Extraction methods should be selective, cost-effective, reproducible, environmentally sustainable, safe, and efficient [132]. Techniques for obtaining natural bioactive compounds are generally categorized into conventional and modern approaches (Figure 1). Conventional methods include maceration, percolation, decoction, reflux, and Soxhlet extraction, which commonly employ solvents such as water or organic solvents like methanol, ethanol, propanol, acetone, and ethyl acetate [132,133]. These methods have disadvantages such as non-selective extraction and the potential degradation or isomerization of bioactive compounds caused by prolonged heating, along with low extraction efficiency, extended processing time, and the high usage and cost of organic solvents and problems related to solvent recycling, which necessitate the development of other environmentally friendly techniques [132,133,134].

Extraction of bioactive compounds by conventional methods such as maceration and Soxhlet extraction is limited due to problems such as high solvent consumption, long time, and environmental impacts. Therefore, the development of modern, environmentally friendly, and efficient methods is essential [135].

The so-called modern techniques were developed to reduce the use of toxic solvents, reducing waste and energy consumption, and at the same time increasing the extraction yield [136]. Recently, new methods, including enzyme-assisted extraction, fermentation-assisted extraction, mechanochemical-assisted extraction, ultrasound-assisted extraction, microwave-assisted extraction (MAE), supercritical fluid extraction, and subcritical water extraction, have been proposed [135,136,137]. Such extraction approaches have been used in combination with BET-DES systems for enhancing the extraction efficiency (Table 5). For example, in the extraction of palm oil, the palm oil mixture was combined with BET-NADES at a mass ratio of 1:2 (*w*/*w*) and maintained at 50 °C with stirring at 500 rpm for 3 h. Extraction was performed in a sealed tube to avoid evaporation, and the extracts were subsequently separated by centrifugation at the same temperature [138]. Coumarins from *Angelicae pubescentis* Radix were extracted by BET-based NADES and UAE [139].

BET-glycerol, BET-lactic acid, and BET-levulinic acid were employed for the extraction of phenolic compounds from model coal tar. Findings indicated that phenolic substances could be effectively extracted within a short time using the three DESs. Among them, BET–glycerol was identified as the most suitable solvent, exhibiting the highest extraction efficiency of 94.6% and 5.2% neutral oil entrainment under optimal conditions [142]. 5-(Hydroxymethyl)furfural (HMF) represents an important platform chemical obtained from carbohydrates such as fructose and sucrose. Its efficient production is crucial for the development of sustainable bio-based chemicals and fuels. The MAE reactions in the presence of BET-based NADES significantly accelerated the conversion of fructose and sucrose into HMF. The use of NADES containing carboxylic acids as HBD and water significantly enhanced the yield of HMF, achieving up to 94% from fructose and 72% from sucrose, notably reducing the reaction time from 45 min to just 11 min [141].

The NADES, composed of BET and polyalcohol as a green alternative to steam-stripping, was used in palm oil refining and reducing free fatty acids (FFAs), particularly palmitic acid. Traditional steam-stripping removes FFAs but also degrades valuable nutraceuticals (e.g., tocopherols and carotenoids) and consumes significant energy. The study evaluated the viscosity of the tested NADES, which ranged from 10–236 cSt, while polarity was 48.9–50.8 kcal/mol. Higher viscosity reduced the extraction efficiency when NADES had a similar polarity to BET. The best-performing NADES (BET with 1, 2-butanediol) achieved a 60% (*w*/*w*) extraction yield. The results suggest that NADES can effectively extract FFAs while preserving nutraceuticals and reducing energy use, making them a promising, eco-friendly solvent for palm oil refining [138].

BET has also been used as a halide ion entity for the preparation of DES and for extracting phenolic compounds from SCG. The study highlighted the potential of SCG, typically regarded as waste, to yield valuable bioactive compounds known for their antioxidant and health benefits. Results indicated that the BET-1,2-butanediol combination exhibited the highest total phenolic content and strong antioxidant properties [10].

Coffee beans contain several compounds, including chlorogenic acids (CGAs), which have demonstrated nutraceutical properties; notably, CGA molecules are also found in the waste by-product known as SCG. A total of fifteen different DESs, formulated with ChCl and BET as HBAs in combination with seven distinct alcohols, two organic acids, sugar, and urea, as HBD, were tested for the extraction of CGAs from SCG. Among them, the BET-based DES combined with triethylene glycol at a 1:2 molar ratio proved to be the most effective, outperforming even the conventional organic solvent (methanol/H_2_O, 70:30) [140].

ATPS, composed of two DESs, including ChCl/urea and BET/urea, in combination with dipotassium hydrogen phosphate, were employed for the extraction of hexavalent chromium. Extraction efficiency improved with increasing temperature, metal concentration, and salt content. The BET/urea system achieved an extraction efficiency of ~89.51 ± 1.69 at pH 8.9, 25 °C, and a metal concentration of 80 mg/L. Given the comparable cost of both systems, BET/urea was determined to be the more effective option for chromium separation [144].

The MAE-DES method was proposed to extract active compounds from peony petals. Four different components of DESs were applied to the measurement of total phenolic content (TPC), total flavonoid content (TFC), and total anthocyanin content (TAC). The results indicated that, under optimized conditions, the extraction yields of TPC, TFC, and TAC were 321.59 mg GAE/g, 61.65 mg RE/g, and 2.15 mg C_3_GE/g, respectively. The extraction mechanism of DESs for active compounds was further investigated using density functional theory, and molecular dynamics (MD) simulations were conducted. The results demonstrated that the extraction efficiency of active compounds was influenced by the hydrogen-bond interactions with DES [145].

BET: urea-DES was used as the separation medium in capillary electrophoresis (CE), as a widely used analytical method. This solvent has desirable properties such as suitable viscosity, thermal stability, and UV compatibility. BET: urea-DES can separate various compounds (cationic, neutral, and anionic) well in capillary zone electrophoresis. Also, by adding sodium dodecyl sulfate, a micellar electrokinetic chromatography system was developed that is able to separate similar neutral compounds that could not be separated in conventional aqueous media [146].

Orange peels are considered a major agro-industrial residue from orange fruit processing. The BET and glycerol-based NADES were evaluated for phenolic compounds extraction and hydro-distillation of orange peels, employing an UAE technique. The results demonstrated that combining NADES with ultrasound facilitates greener and safer processes by reducing reagents, waste generation, and energy consumption [147].

BET-urea DESs in ATPS enable efficient protein extraction (~99.82%) with minimal conformational changes, preserving protein functionality. These systems also allow for back-extraction and maintain protein stability, proving practical for biotechnological and pharmaceutical applications. BET-urea DES has demonstrated exceptionally high protein extraction efficiency (~99.82%) in green ATPS [116]. This system preserves the native conformation of proteins, minimizing structural changes during extraction, which is critical for maintaining protein functionality in biotechnological applications. Back extraction and stability studies confirm the practical applicability of this DES-based ATPS for protein separation and purification, highlighting its potential in sustainable bioprocessing.

BET hydrochloride-based DESs have been explored for metal extraction and electrodeposition in ion metallurgy. Although promising for the rapid dissolution of metal oxides, challenges remain due to solvent decomposition reactions affecting environmental friendliness and usability [16].

BET-based DES offers notable advantages over conventional ChCl–based DESs, which commonly contain halogen atoms. Although the extraction efficiency of BET-Gly is reported to be approximately 4% lower than that of the ChCl–Gly system (98.3% extraction efficiency with 4.2% neutral oil entrainment), the absence of halide ions in Bet-Gly and its non-corrosive behavior toward steel equipment provide a fundamental and irreplaceable advantage compared with choline-derived solvents. This aspect is particularly important in industrial applications, where the presence of chloride in ChCl-based DESs can lead to severe equipment corrosion. Given these characteristics, Bet-Gly can be considered not only an efficient, environmentally friendly, and cost-effective solvent for phenol extraction, but also a BET-based DES with significant operational and corrosion-resistance benefits over choline-based systems [142].

### 6.2. Catalysis and Chemical Synthesis (e.g., Organic Reactions, Enzymatic Processes)

DESs have demonstrated catalytic activity in various organic reactions, including addition, cyclization, replacement, multicomponent, condensation, oxidation, and reduction reactions [148] (Table 6). BET-based DESs have been employed as reaction media for biocatalytic processes, improving selectivity and sustainability in organic synthesis. For example, recombinant *E. coli* cells achieved high yields and enantioselectivity in the reduction of aromatic ketones when BET-based DESs were used as solvents [149]. The impact of BET-based NADESs on enzymatic reactions has been characterized by assessing enzyme activity and stability. Studies indicate that certain DES formulations can significantly enhance enzymatic activity; up to two-fold increases were observed with specific combinations [114,121]. DESs, particularly those based on ChCl or BET, have shown promise in biocatalytic reactions, enhancing enzyme stability and efficiency while acting as extraction solvents, reaction media, and even substrate sources [150]. Their applications span organo-catalysis, bio-transformations, and multistep processes [151]. Recent research has explored DESs in homogeneous, heterogeneous, and electrocatalytic reactions for organic synthesis, biomass conversion, and pharmaceutical manufacturing [152]. BET: lactic acid NADES (1:2 molar ratios) has demonstrated effective catalytic performance over multiple cycles in organic synthesis. Additionally, BET-based DESs serve as dual-functional solvents and reactants in slurry synthesis methods for cocrystal production, eliminating the need for organic solvents and enabling control over molecular interactions by adjusting water content or DES ratios [19].

In the bioconversion of ginseng extracts into deglycosylated ginsenosides using BET-based DES, DES not only enhanced the enzyme activity of β-glucosidase and β-galactosidase but also improved the conversion of ginseng extracts into more bioavailable ginsenosides, while maintaining stability across different pH and temperature conditions. Results revealed that the DES composed of BET and ethylene glycol (1:2 molar ratio) significantly promoted the activity of the enzyme combination and enhanced both acid resistance and thermal stability [153]. BET-based DES has also shown promise as an efficient medium for biocatalysis, enabling the synthesis of antimicrobial polymers without toxic solvents [100,114].

Non-productive oil palm trunks (OPTs), rich in starch and cellulose, are promising raw materials for bioethanol production. Effective pre-treatment is essential to optimize the cellulose-to-lignin ratio, facilitating hydrolysis. The delignification efficiency of NADES on OPT, using a 1:2 ratio of BET/lactic acid and BET/glycerol, achieved optimal delignification, reducing lignin content by 26.73% and 26.46%, respectively, over 2 h at 100 °C. These results indicate that both BET/lactic acid and BET/glycerol are natural solvents with strong potential for lignin breakdown in non-productive oil palm trunks. The findings suggest that BET-based NADES could be effective in improving bioethanol production from oil palm trunks through efficient delignification [156].

To improve the enzymatic digestibility of wheat straw, N-(2-hydroxyethyl) ethylenediamine (NE) was chosen as the HBD for preparation of a BET-based DES. The BET: NE mixture was combined with KOH to pre-treat wheat straw at 100 °C for 50 min. This KOH/BET: NE system efficiently removed lignin and hemicellulose while retaining a substantial portion of cellulose, achieving 95.2% delignification and 90.8% cellulose retention. Relative to untreated wheat straw, the saccharification activity of DES-treated straw increased 5.1-fold, yielding a final reducing sugar concentration of 98.3%. After pre-treatment, wheat straw accessibility rose to 340.1 mg/g, while hydrophobicity and lignin surface area decreased to 1.6 L/g and 108.6 m^2^/g, respectively. Significant changes in surface morphology were observed, indicating enhanced enzymatic hydrolysis. In conclusion, KOH/BET: NE pre-treatment effectively disrupted the crystalline structure of wheat straw, removing lignin and hemicellulose and thereby improving enzymatic hydrolysis. This alkali-assisted BET-based DES system represents an efficient approach for biorefining [157].

Reports have shown that BET-glycerol DES at a 1:2 molar ratio significantly enhances the thermal stability of enzymes such as laccase compared to choline-based DES analogs. Specifically, laccase retains about 60% of its activity after 24 h in DES buffer mixtures containing 35 wt% water, demonstrating notable enzyme protection under thermal stress [121]. BET-glycerol DES stabilizes enzyme structure, likely due to strong hydrogen bonding and electrostatic interactions within the DES, which protect the enzyme from denaturation at elevated temperatures. The viscosity of BET-based DESs, including systems like BET-1,2-propanediol, shows a strong temperature dependence following Arrhenius behavior. This means viscosity decreases exponentially with increasing temperature, influencing enzyme mobility and reaction kinetics [158]. The combination of enhanced enzyme stability and tunable viscosity at moderate temperatures makes BET-based DESs promising media for industrial enzymatic processes, improving enzyme lifetime and efficiency while allowing control over reaction conditions.

In thermal stability, BET-glycerol DES (1:2 ratio) enhances enzyme stability compared to choline-based analogs, with laccase retaining ~60% activity after 24 h in DES-buffer mixtures containing 35 wt% water [121]. However, viscosity exhibits strong temperature dependence, following Arrhenius behavior in systems like BET-1,2-propanediol. This necessitates precise thermal control in industrial processes, as high temperatures (80–90 °C) improve fluidity but risk destabilizing the hydrogen-bonded network [121,159]. The balance between thermal optimization and solvent integrity remains critical for applications requiring both high-temperature operation and structural consistency.

Nikodajevic et al. [160], employed a mixture of BET hydrochloride, glycerol, and urea as both a dye carrier and a fabric surface modifier. The use of DES enhanced the dyeing efficiency, eliminating the need for auxiliary agents such as carriers and dispersing agents that are harmful to the environment. Furthermore, a more hydrophobic type of this DES was also utilized for dyeing polyester fabrics.

### 6.3. Specific Applications in Pharmaceutical Sciences

DESs enhance the solubility and stability of bioactive compounds, which is crucial for pharmaceutical applications. It has been reported that BET-based DES can increase the solubility of small molecules like rutin by 50–100 times compared to water, making them valuable for drug formulation and delivery systems [135]. Liu et al. screened twenty-seven DESs in combination with UAE to extract scutellarin from *Erigerontis herba*, a traditional Chinese medicinal herb. Among the DESs tested, twenty-five demonstrated higher extraction yields compared to conventional solvents such as methanol and a 75% ethanol solution [161]. Beta-lactam antibiotics are highly unstable in aqueous media, potentially resulting in subtherapeutic concentrations, antimicrobial resistance, and treatment failure. Belén Olivares et al. demonstrated that a NADES composed of BET and urea can enhance the stability of certain beta-lactams, including imipenem, the most unstable antibiotic in this class [162]. *L*-ascorbic acid (AA), a powerful antioxidant, is widely used in pharmaceutical and cosmetic topical formulations. However, its application is limited due to instability and poor skin permeability. One study proposed enhancing AA solubility and topical delivery by converting it into a therapeutic deep eutectic system (THEDES). AA and BET-based THEDESs were prepared at specific molar ratios and characterized using polarized optical microscopy, Fourier transform infrared spectroscopy, and differential scanning calorimetry. Solubility tests demonstrated that AA in the form of THEDES was highly soluble (≈40%) in various polyols, including glycerin, 1,3-butylene glycol, dipropylene glycol, and 1,3-propanediol. These results highlight the potential of AA and BET-based THEDES as novel transdermal delivery systems. Moreover, this approach was successfully extended to gluconolactone, another natural antioxidant, indicating its broader applicability for transdermal delivery [115].

The synergistic interaction between BET and urea was evaluated when BET and urea were combined at a specific molar ratio ranging from 1:1 to 1:3, resulting in a liquid despite both substances being solid at room temperature. This research highlighted the importance of understanding how these compounds work together, especially regarding their role in protecting proteins from denaturation caused by urea. They could not determine the freezing points of several BET/urea mixtures; instead, glass transitions were observed at approximately −50 °C [163]. Cardellini et al. investigated the formation of DESs by combining BET with 23 aromatic carboxylic acids and 13 aliphatic carboxylic acids. These zwitterionic DESs remain liquid at room temperature or below 70 °C and, importantly, contain no chloride or metal ions, making them suitable for diverse applications due to their low melting point (−36 °C). Given these advantages, the solubility of several α-*L*-amino acids was examined in these DES, showing particularly good solubility for aromatic amino acids, which typically have poor solubility in water [164]. The green extraction of chlorogenic acid (CGA) from *Lonicera japonica* Thunb. Using DESs was systematically studied. BET-glycerol exhibited superior extraction efficiency and was selected for process optimization using a Box–Behnken Design. Under optimized conditions, the CGA yield was 1.9 times higher than that achieved with ethanol extraction. Molecular simulations were performed to investigate the mechanistic basis for the differences among BET-based DESs, revealing that higher CGA yields were associated with increased interaction energies and a greater number of hydrogen bonds. These results emphasize the importance of identifying key interaction sites in both target molecules and extractants to rationally design tailored solvents for improved extraction performance [165]. The solubility profiles of selected active pharmaceutical ingredients in ChCl- and BET-based DESs were investigated using machine-learning-based theoretical models. Comprehensive analysis showed that enlarging the dataset and testing a variety of solvent combinations substantially improves the predictive accuracy of the models [166]. BET, a bio-based surfactant, is known to enhance drug absorption, protect drugs from degradation, and improve the efficacy of therapeutic and hygiene products. To study the interactions between gabapentin (an antiepileptic drug) and different BET-based compounds, including BET, BET octyl ester chloride ionic liquid, and BET-urea (1:2 molar ratio), experiments were performed at 298 K. The analysis of apparent specific volume (ASV) and hydration number (nH) of gabapentin in these systems revealed that gabapentin exhibits a bitter taste in aqueous DES solutions and becomes most dehydrated in the presence of BET [167]. The key findings of some studies on BET-based DESs in pharmaceutical applications are summarized in Table 7.

### 6.4. Specific Applications in Food Science and Preservation

BET-based DESs have shown significant potential in food preservation (Table 8). These solvents enhance the stability of extracted pigments, making them suitable for use as natural food colorants with extended shelf life compared to traditional methods [168,169]. BET DESs also outperform conventional solvents like ethanol in extracting nutraceuticals from coffee grounds and proteins from biological materials [117,170]. BET-based DESs as green solvents were proven effective for extracting valuable bioactive compounds from SCG [10]. Vinci et al. employed DESs to extract antioxidant compounds from dark chocolate. The BET–ChCl DES achieved an extraction yield 35% higher than that obtained with conventional solvents [171]. Oleanolic acid is the main component of wine pomace and has anti-caries, antitumor, and anti-obesity effects, and protects hepatic function. BET-based DESs were used for the efficient extraction of oleanolic acid from wine pomace. The result indicated that the extraction of pomace using BET-xylitol (molar ratio 3:2) afforded the highest yield of oleanolic acid among DESs [172].

Antioxidant and naturally occurring molecules can act as either HBDs or HBAs to form natural NADES, which possess inherent antioxidant activity. Consequently, DESs incorporating extracted nanoparticles (NPs) can produce synergistic antioxidant effects, making the system a promising active ingredient for food industry applications. This synergy results from improved solubilization and stabilization of NPs in DESs through hydrogen bonding, ionic interactions, and van der Waals forces. Together, the DES–NP system effectively neutralizes oxidants such as ROS and free radicals and modulates their reactions with other molecules, achieving up to a threefold increase in activity compared to extracts in conventional solvents in vitro [173].

Eutectogels, a family of emerging materials, integrate the characteristics of DES and gels, presenting unique application potential in biomedical engineering [176]. DES-based eutectogels combine the benefits of DESs’ ionic properties with gel-like mechanical features, offering enhanced conductivity and chemical tunability. Preparation of DES-based eutectogels generally involves the addition of gelators or polymers [177]. For example, a small amount of a gelator (e.g., 1,3:2,4-dibenzylidene-*D*-sorbitol, DBS) or polymer (e.g., polyvinyl alcohol, PVA, Carbopol microgels) is added to the DES. The gelator or polymer forms a network structure via non-covalent interactions (hydrogen bonding, van der Waals forces) that immobilizes the DES, transforming it into a gel-like material known as eutectogels. Key characteristics of the resulting eutectogels include gel-like rheology, high ionic conductivity (comparable to the original DES), thermal stability, and potential self-healing behavior. These features make eutectogels attractive for applications in energy storage, extraction, and biomedical fields [178]. Recently, DESs composed of BET and xylitol were simultaneously employed as a green medium for the synthesis of Au NPs and incorporated into PVA-based eutectogels. Both the pristine eutectogel and the AuNP-integrated composite exhibited favorable functional properties, including pH-dependent stability and absorptive capacity [179].

Wan and co-workers developed gelatin films plasticized with two different DESs for moisture-regulating cherry tomatoes (*Solanum lycopersicum* L.). The tested DESs were ChCl-glycerol and BET-glycerol. After 9 days, the degree of spoilage was reduced significantly for DES-containing film [174]. DESs are being explored for the synthesis of biodegradable food packaging materials, offering an eco-friendly alternative to plastic packaging while maintaining food quality [170]. BET-based DES containing lactic acid has been used as a plasticizer in food packaging [180]. Grapes are among the most widely cultivated fruit crops, and grape pomace is the main solid by-product obtained in the wine-making process. For the extraction of bioactive molecules of grape pomace, the seven selected DESs were used. Significant antibacterial effects were observed for the BET/lactic acid 1:4 DES extract with 40% water originating from different phenolic compounds, including three quercetin glucosides [175].

BET-based DESs with different BET: urea molar ratios (1:2, 1:3, 1:4, and 1:5) were evaluated as bio-plasticizers for thermoplastic starch (TPS) obtained by thermal compression. TPS obtained from urea-rich BET-DES showed a favorable plasticizing performance [181].

Films fabricated using BET-based DES combined with oxalic acid to produce zwitterionic cellulose nanofibers (Z-CNFs) displayed desirable optical transmittance, high haze, and remarkable tensile strength. In this approach, BET introduces quaternary ammonium groups and oxalic acid provides carboxyl groups, playing the central role in modifying the cellulose surface and imparting zwitterionic behavior. The resulting Z-CNFs preserved the cellulose Iβ crystalline structure, exhibited good thermal stability, and showed shear-thinning rheological properties [182].

DESs have demonstrated potential in cryopreservation, where they protect food components during freezing by stabilizing enzymes and biomolecules. For instance, BET-sorbitol DES improved the thermal stability of horseradish peroxidase, which is relevant for enzymatic food preservation [113,114]. They can also act as antioxidants, reducing oxidative degradation in stored food [14]. BET-based DESs are used in enzyme-catalyzed reactions to improve enzyme stability under harsh conditions (e.g., varying pH or temperature), enhancing reaction efficiency in food processing [114,121]. These systems have achieved remarkable efficiencies, such as 99.8% protein extraction in aqueous two-phase systems, reducing the need for expensive reagents and simplifying workflows [17,18].

### 6.5. Specific Applications in Electrochemical Energy Storage

BET-based NADESs are being developed as sustainable electrolytes for energy storage devices such as supercapacitors, batteries, and accumulators. For example, NADESs composed of BET hydrochloride as HBA and ethylene glycol as HBD exhibit low viscosity, high ionic conductivity, and electrochemical stability, rendering them suitable for long-term use in supercapacitors with high specific capacitance and stability [183]. BET-based DESs were synthesized with urea, lactic acid, glycerol, 1,2-propanediol, and xylitol. Thermogravimetric analysis showed that BET-xylitol and BET-urea were thermally stable (>150 °C). Glycerol- and 1,2-propanediol-based DESs showed the greatest surface tension values. BET-urea and BET-lactic acid showed high conductivity [110].

### 6.6. Specific Application in the Environment

CO_2_ is a major greenhouse gas, contributing significantly to global warming and climate change due to human activities such as fossil fuel combustion, industrial processes, and deforestation. Efficient CO_2_ capture technologies are critical for mitigating the adverse effects of air pollution. Based on reports, BET-based DESs exhibit a superior capacity for CO_2_ absorption compared to their ChCl-based counterparts. For example, BET-Methyl diethanolamine in a 1:6 ratio demonstrated the highest CO_2_ absorption at 313.15 K and 6 bar [184]. Ternary formulations of BET-based DESs, including combinations of diethanolamine and ethylene glycol, have shown CO_2_ absorption capacities reaching up to 0.207 g CO_2_/g DES under optimal conditions, outperforming many systems based on ChCl [185]. One notable study reported an impressive CO_2_ uptake of 0.0319 mol of CO_2_ per mol of DES using a specific formulation in conjunction with a superbase [186].

The enhanced performance is attributed to the molecular structure and interactions within BET-based NADESs, which reduce viscosity and mass-transfer resistance, allowing for more efficient CO_2_ capture [184,186]. By removing CO_2_ from emissions before they reach the atmosphere, these technologies help reduce the greenhouse effect and slow global temperature rise. BET-based DESs in glycerol-BET-water systems have exhibited non-linear structural transformations depending on the water content. Specifically, at water concentrations between approximately 29.5 and 54.4 wt%, the hydrogen-bonding networks between BET and glycerol break down, causing a transition from a DES-dominated structure to one that behaves more like an aqueous solution [159,187]. This disruption compromises solvent integrity in humid environments, with water molecules replacing glycerol around the BET and altering diffusion rates. For applications like CO_2_ capture, water absorption reduces capacity by ~15% after five cycles due to weakened intermolecular interactions [159].

In BET-based DESs, particularly the BET/ethylene glycol (1:3) formulation, have demonstrated high efficiency in removing nitrogen- and sulfur-containing pollutants such as pyridine (99.7% removal) and thiophene (57.5% removal) from petroleum fractions like n-heptane [188]. This is a significant advancement for environmental pollution control in the petroleum industry due as sulfur and nitrogen compounds in fuels are major contributors to air pollution when combusted, releasing harmful gases such as sulfur oxides and nitrogen oxides. These gases cause acid rain, respiratory problems, and contribute to smog formation. Effective desulfurization and denitrogenation reduce these emissions at the source by purifying fuels before combustion, thus mitigating their environmental and health impacts. The high selectivity and capacity of BET/ethylene glycol DESs improve the efficiency of removing these pollutants, potentially lowering the energy and chemical consumption of purification processes.

BET-based DESs operate effectively at moderate temperatures between 60–80 °C during biomass pretreatment processes, which is significantly lower than many conventional methods that often require temperatures above 100 °C. This lower operational temperature translates into reduced energy consumption, making these solvents more energy-efficient and sustainable for industrial applications [17,135,189]. Compared to traditional pretreatment methods such as steam explosion or alkaline treatments, which often operate at higher temperatures (e.g., 120–150 °C), BET-based DESs enable efficient delignification and biomass fractionation at milder conditions, preserving valuable biomass components while saving energy [190]. The moderate temperature operation also minimizes thermal degradation of sensitive biomass components, enhancing the quality and yield of downstream products like fermentable sugars and lignin valorization. Their adaptability to industrial-scale processes is evident in applications like biorefineries for bioethanol production and material synthesis for conductive polymers. This scalability underscores their potential to revolutionize industries by offering greener and more cost-effective alternatives to traditional solvents [18].

According to the findings of Liu et al.’s study on CO_2_ capture using ternary BET-based DESs, the main limitations hindering their effective industrial application include low regenerability, high water sensitivity, and challenges related to viscosity. The presence of water significantly reduces the performance by disrupting hydrogen bond networks, lowering CO_2_ absorption capacity, and interfering with active sites. Water content also affects their stability and reactivity in certain applications. While BET-based DESs can be regenerated, residual CO_2_ remains due to the formation of stable carbamates during chemical absorption, leading to a gradual decrease in absorption capacity over cycles [185]. Some BET-based DESs exhibit much higher viscosity than their individual components, particularly at lower temperatures, which can hinder mass transfer during processes like CO_2_ capture or separations [117,185]. Chen et al. (2020) [158] reported that although viscosity in BET-based DESs decreases with increasing temperature and higher HBD ratios, it remains a significant operational challenge at ambient conditions. Surface tension is consistently higher than that of pure 1,2-propanediol, potentially hindering interfacial processes such as emulsification, extraction, and catalysis. The study also revealed compositional rigidity, as attempts to prepare DESs at lower HBD ratios (1:2, 1:3) failed to produce homogeneous mixtures, restricting formulation flexibility. Moreover, while viscosity trends were modeled using the Arrhenius equation, substantial deviations from experimental data, especially across wider temperature ranges, highlight limitations in predictive accuracy and scale-up reliability [117].

Their polarity may limit their compatibility with certain solutes or reactions [3]. Binary systems (e.g., BET-ethylene glycol) rely on physical absorption mechanisms for CO_2_ capture, which are less effective compared to ternary systems involving chemical absorption. Moreover, BET-based DESs, despite their metal dissolution capabilities, suffer from poor thermal and chemical stability, leading to decomposition even at moderate temperatures. These reactions produce toxic by-products, hinder recyclability, and alter solvent composition. Additionally, hygroscopic behavior and crystallization at room temperature further limit their industrial viability [14]. In summary, while BET-based DESs are promising for sustainable applications due to their eco-friendliness and versatility, challenges such as water sensitivity, viscosity issues, and limited regenerability must be addressed for broader adoption.

**Figure 2:**
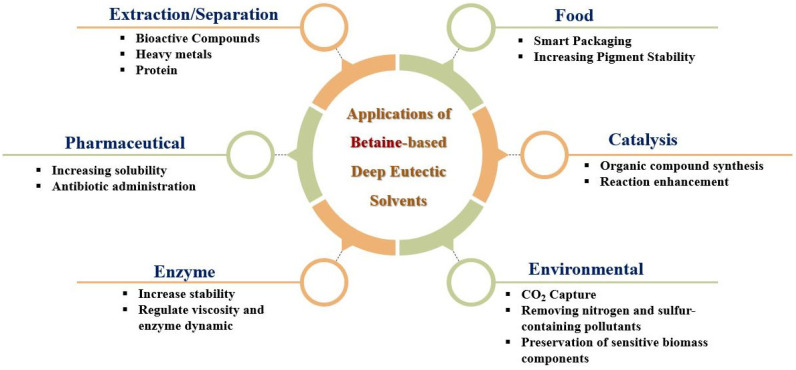
Summary of various Betaine (BET)-based DES applications.

Figure 2 provides an overview of the applications of BET-based DES in various scientific and industrial fields, highlighting their importance as a green and efficient alternative to traditional organic solvents.

## 7. Regulatory and Safety Considerations

In most studies, DESs have been referred to as green solvents, primarily because their components are abundantly found in nature, for example, choline and betaine, both of which are found abundantly in food sources as essential nutrients and exhibit lower volatility compared to organic solvents, which are mostly volatile and lipophilic [191]. Recent study results have shown that these solvents can also have degrees of toxicity due to the diversity of HBAs and HBDs and possible interactions. Therefore, each should be examined separately. Therefore, recent studies have studied the toxicity and biodegradability of specific DESs and NADES using different protocols. For example, some toxicological studies were conducted using ChCl-based DESs on bacteria [192,193,194] and other microorganisms [195,196], animals (both in vivo and in vitro studies) [194,195,197,198,199], and plants [200,201]. Choline chloride and its esters, including choline chloride itself, are banned from use in cosmetic products under European regulations [202].

The number of toxicity studies specifically on BET and BET-based solvents is limited, but some notable findings are available. The sub-acute and sub-chronic toxicology studies of BET in rats showed that even high dietary doses of BET (up to 5%) were essentially nontoxic [203].

The BET-urea DES (BU) demonstrated a favorable toxicity profile across multiple biocompatibility assessments. Cytotoxicity testing on primary human dermal fibroblasts revealed a high IC_50_ value of 75 mg/mL, indicating low toxicity and suggesting that BU is significantly less cytotoxic than many ammonium-based DESs, having choline chloride as the hydrogen acceptor, and ionic liquids, which often exhibit toxicity in the micromolar or millimolar range. This low cytotoxicity is attributed to BU’s non-ionic, zwitterionic character and the absence of strong cationic surfactancy, which reduces membrane disruption. Immunogenicity assays using THP-1-derived macrophages showed no induction of pro-inflammatory cytokines (IL-6, IL-8, TNF-α) following BU exposure, confirming its non-immunogenic nature. Furthermore, in an ex vivo human skin explant model, BU application resulted in no significant histopathological damage after 24 and 48 h, with no signs of irritation, hyperplasia, apoptosis, or inflammation observed. This absence of adverse effects, despite BU’s high urea content, is likely due to the strong hydrogen-bonding network between betaine and urea, which reduces the availability of free urea and mitigates its potential irritant properties [162].

A study on a NADES composed of BET and glycerol (1:2 mole ratio) showed toxic effects in rats when administered orally over 14 days. It caused mortality in two out of six rats, along with signs like excessive water consumption, reduced food intake, weight loss, hepatomegaly, oxidative stress, and lipid abnormalities. This indicates potential toxicity concerns with oral exposure under the studied conditions, despite the solvent containing bioactive polyphenols [204]. In contrast, another study evaluating BET-based DES toxicity in different biological models, such as zebrafish subjected to intraperitoneal injections, reported no significant toxicity even at relatively high concentrations (e.g., 3000 µM for BET: glycerol DES and 5000 µM for BET: sorbitol: water DES) [14].

According to the results of Rodrigues et al.’s study, BET/polyol DESs (BET: glycerol, BET: propylene glycol, BET: ethylene glycol) generally exhibited a low toxicity profile across different biological models, with strong dependence on composition and water content. In human intestinal epithelial cells (Caco-2), DES exhibited limited cytotoxicity, which decreased further upon addition of water, thereby enhancing cell viability. Antimicrobial assays revealed only weak to moderate inhibitory activity against *Staphylococcus aureus* and *Escherichia coli*, which further decreased upon dilution with water. Similarly, phytotoxicity tests on wheat seeds indicated minimal inhibition of germination and growth, with biochemical markers such as lipid peroxidation, chlorophyll levels, and antioxidant enzyme activities reflecting only mild stress responses at higher concentrations. Generally, these systems are safe at moderate concentrations and under hydrated conditions, exhibiting low cytotoxicity in human intestinal cells along with limited antimicrobial and phytotoxic activity. In contrast, highly concentrated and viscous formulations can trigger biological stress responses, particularly in plants, underscoring the importance of carefully optimizing both composition and water content [1]. These studies also indicated an absence of major adverse effects on antioxidant enzyme activities or lipid peroxidation, suggesting good biocompatibility and environmental safety for these solvents within specific concentration limits [1,14]. BET-based DESs did not significantly affect enzymatic activity or oxidative stress markers, supporting their biocompatibility [14].

Overall, these results highlight the promise of BET-based DESs as environmentally friendly solvents, while also stressing the necessity of dose-dependent and application-specific safety evaluations before their adoption in industrial or food-related contexts.

The regulatory landscape for BET-based DES is evolving as its applications expand. Regulatory bodies are increasingly focusing on the environmental impact and safety profiles of new solvent systems. In Europe, for instance, regulations under REACH (Registration, Evaluation, Authorisation, and Restriction of Chemicals) require comprehensive data on chemical substances, including their toxicity and environmental effects. This necessitates that manufacturers provide detailed safety data sheets and conduct thorough risk assessments before market introduction [205].

## 8. Future Perspectives

As interest in BET-DESs grows, several key research directions and challenges must be addressed for their broader industrial application. Research should focus on optimizing formulations by varying hydrogen bond donors, acceptors, molar ratios, and additives to improve efficiency and selectivity in extracting bioactive compounds.

Specifically, J. Richter et al. found that BET-based DESs undergo numerous decomposition reactions even at relatively low temperatures (60 °C), making repeated recycling “not conceivable”. The researchers identified that the decomposition products not only complicate solvent reuse but also potentially introduce toxicity concerns [16]. In-depth mechanistic studies using advanced analytical techniques are necessary to understand BET-DES interactions with target compounds. Dinis O. Abranches et al. further highlighted the complex molecular interactions of BET, noting its “strong negative deviations from ideality” when mixed with different organic substances [100].

Scaling up production through cost-effective and energy-efficient methods is essential for commercial viability. Comprehensive toxicological and environmental impact assessments are needed for regulatory approval and public acceptance. BET-DES also holds potential in renewable energy applications like CO_2_ capture and biomass conversion. Their high viscosity and density pose challenges for fluidity and mass transfer, which can be mitigated by formulation adjustments. Leon Meredith et al. additionally demonstrated that even small water additions can dramatically alter the solvent’s physico-chemical properties, suggesting inherent instability [206]. These findings indicate that while BET-based DESs are innovative, substantial research is needed to overcome their current limitations.

Interdisciplinary collaboration can foster innovation in novel extraction techniques. Studies on stability and reuse will inform industrial applicability. Finally, establishing regulatory frameworks early will facilitate smoother commercialization pathways. Addressing these challenges will unlock the full potential of BET-DES as a sustainable green solvent across multiple industries.

## 9. Conclusions

With increasing industry emphasis on sustainability, BET-based DESs are well-positioned as next-generation green solvents. BET acts predominantly as a strong hydrogen bond acceptor, especially because of polarity imbalances and the presence of the carboxylate group. BET-DESs offer effective solvent systems with wide-ranging applications in extraction, pharmaceuticals, food science, and catalysis. BET-DESs also enable tailored solvent properties through variation of hydrogen bond donors and acceptors. They provide excellent solubilization and ex-traction efficiency for a wide range of bioactive compounds, including phenolics, antioxidants, flavonoids, and polysaccharides from plant and biological materials. Overall, while BET-based DES offers many advantages, careful control of viscosity and regeneration, along with sensitivity to water, is necessary to overcome these limitations effectively. However, further studies are needed to understand structure-activity relationships to select optimal BET-DESs for specific applications. Comprehensive research on extraction techniques and solvent reuse will be essential to fully realize their industrial potential. Continued innovation and evaluation are warranted for sustainable alternatives to conventional toxic solvents, advancing environmentally responsible practices across multiple industries.

## Figures and Tables

**Table 1:** The Main Properties of BET [8,9].

Scientific Name	Trimethyl Glycine
Chemical Formula	C_5_H_11_NO_2_
Molar Weight	117.15 g/mol
Natural Resources	Beets (*Beta vulgaris*), wheat bran, wheat germ, spinach, microorganisms, aquatic invertebrates
Color	White hygroscopic crystals
Taste	Sweet
pKa	1.83 at 0 °C
Melting Point	293 °C (decomposes)
Solubility (g/100 g solvent)	Water: 160 Methanol: 57 Ethanol: 8.7
Aqueous Solution	Clear and colorless

**Table 2:** Common Foods Rich in BET and Their Approximate BET Content per 100 g.

Food Item	BET Content (mg/100 g)	Food Item	BET Content (mg/100 g)
Wheat Bran	1200–1300	Rye Bread	250–400
Wheat Germ	~1200	Whole Wheat Bread	150–400
Spinach (raw)	600–645	Pasta (whole wheat)	100–250
Beets (raw)	175–300 (Up to 750 µg/g dry wt)	Shrimp	1000–2000
Quinoa (cooked)	150–200	Clams	200–900
Mussels	1100–2000	Sweet Potato	35–50
Oysters	400–900	Turkey Breast	45–75
Veal	60–80	Beef	20–50

**Table 3:** Principal Chemical and Physiological Roles of BET.

Fields	Applications	References
Chemical	- Osmolyte in cell volume regulation	[75]
	- Stabilizes protein/DNA as a chemical chaperone	[76]
	- PCR enhancer	[77]
	- Cold acclimation in the plant	[78]
	- Cryoprotectant for microorganism storage	[79]
	- Surfactant	[62]
Metabolic	- Methyl donor in the methionine cycle	[80]
	- Activation of hepatic AMP-activated protein kinase	[81]
	- Preservation of mitochondrial function	[82]
	- Improves insulin sensitivity of adipose tissue	[83]
Clinical Medicine	- Decreases plasma homocysteine in patients with homocystinuria	[84]
	- Promotes viability and development of mouse blastocysts	[85]
	- Reduces injury and improves liver function in animal models with liver disease involving fatty liver. The human application remains speculative	[8]
Agriculture	- Increase lean muscle mass in livestock (e.g., pigs, poultry)	[86]
	- Salmon farming	[87]

**Table 4:** Efficiency Comparison of different BET extraction methods.

Method	Extraction Efficiency (%)	Stripping/Recovery (%)	Purity (%)	Key Advantages
Reactive Extraction (DNNDSA)	67–71	47–54	Moderate	High efficiency, easy, economical
Alcohol Extraction + Crystallization	~40–45 (dry matter)	Not specified	High	Reduced acid use, avoids impurities
Chromatographic Separation	>80	Not specified	~99.8	High yield, high purity, industrial
Membrane Technology	66–91 (stripping)	66–91	Moderate	Continuous, selective, low energy

The symbol ~ indicates an approximate value, the symbol > denotes greater than, and the symbol % represents a percentage.

**Table 5:** The Key Points of Different Research Studies Considered BET-based DESs in Extraction Processes.

DES Components		Molar Ratio	Key Finding/Applications	References
Component 1	Component 2			
BET	1,2-Propanediol	1:3 1:4 1:5	▪ Green extraction of palmitic acid. ▪ NADES, consisting of BET and polyalcohol, are alternative green solvents in the separation of FFAs from palm oil in the solvent extraction process.	[138]
	1,3-Propanediol			
	1,2-Butanediol			
	1,3-Butanediol			
	1,4-Butanediol			
	Ethylene Glycol			
	Glycerol			
BET	1,2-Propanediol	1:3 1:5 1:7	▪ Demonstrating BET-based DESs as green solvents to extract valuable bioactive compounds from Spent Coffee Ground	[10]
	1,3-Propanediol			
	1,2-Butanediol			
	1,3-Butanediol			
	1,4-Butanediol			
	Levulinic acid			
	Lactic acid			
Choline Chloride	Urea	1:2	▪ Green extraction of nutraceutical compounds from Spent Coffee Ground. ▪ Valuable alternative approach to organic solvents for the extraction of nutraceutical compounds from food waste products.	[140]
	Citric acid			
	Lactic acid			
	Glucose			
	Sorbitol			
	Xylitol			
	Glycerol			
	1,6-Hexanediol	1:7		
	Triethylene glycol	1:2		
	Ethylene glycol			
	Propylene glycol			
BET	Lactic acid			
	Glycerol			
	Ethylene glycol			
	Triethylene glycol			
BET Hydrochloride (BHC)	Citric acid: H_2_O	1:1:1	▪ Microwave-assisted HMF production from water-soluble sugars using BET-based NADES ▪ Efficiency of a NADES system and microwave irradiation for dehydration of fructose-based carbohydrates and high HMF yield	[141]
	Tartaric acid: H_2_O			
	Malic acid: H_2_O			
	Lactic acid: H_2_O			
	Glycolic acid: H_2_O			
BET	Glycerol	1:2	▪ Suitability of Bet-Gly for the separation of phenolic compounds from coal tar ▪ Separation of phenolic compounds from oil mixtures by BET-based deep eutectic solvents	[142]
	DL-lactic acid	1:2		
	Levulinic acid	1:2		
BET	Ethylene glycol	1:4	▪ Extraction of coumarins using BET and ethylene glycol-based DES: a new, green, efficient, and cost-effective method ▪ Ultrasonic-assisted extraction of coumarins from *Angelicae pubescentis* Radix by BET-based natural deep eutectic solvents	[139]
	Glycerol	1:2		
BET	Glycerol Glycerol: Water	1:2 1:2:1 1:2:5 1:2:10	▪ Approval of the extract as a final product that can be used as a functional ingredient or natural preservative in the pharmaceutical, nutritional, or cosmetic industries ▪ Unveiling the potential of BET/polyol-based deep eutectic systems for the recovery of bioactive protein derivative-rich extracts from sardine processing residues	[143]
	Propylene glycol Propylene glycol: Water	1:3 1:3:1 1:3:5 1:3:10		
	Ethylene glycol Ethylene glycol: Water	1:3 1:3:1 1:3:5 1:3:10		
BET	Urea	1:2	▪ Separation of heavy metals ▪ Using aqueous two-phase systems based on choline chloride/urea and BET/urea deep eutectic solvents; as a result, the system including BET/urea was evaluated as a better system for the separation of chromium	[144]
	Glycerol	1:1		
BET	Ethylene glycol	1:4	▪ Investigation of BET-based deep eutectic solvent extraction of active compounds from peony petals using molecular simulation and machine learning, and selective extraction of active compounds was verified.	[145]
	1,2-propanediol	1:4		
	Lactic acid	1:2		
	Levulinic acid	1:2		
BET	Urea: H_2_O	1:2:2	▪ BET and urea-based DES as a suitable alternative to traditional solvents and providing a new perspective in designing stable and efficient separation media for CE.	[146]
BET	Lactic acid	1:2	▪ Sustainable extraction of value-added compounds from orange waste using natural deep eutectic solvents and NADES formulated with BET and glycerol (BET-GLY) led to the highest total phenolic content (TPC) among the obtained extracts	[147]
	Glycerol	1:2		
	Ethylene glycol	1:2		
	Urea	1:2		

**Table 6:** The Key Points of Different Research Studies Considered BET-Based DESs in Catalysis Processes.

DES Components		Abbreviation	Molar Ratio	Key Finding/Applications	References
Component 1	Component 2				
Choline Chloride	1,2-Propanediol	ChCl: P	1:2	▪ DES, an efficient and stable transformation medium for converting value-added ginseng extracts to deglycosylated ginsenosides and applicable for green extraction of natural products. ▪ High-value bioconversion of ginseng extracts in BET-based deep eutectic solvents for the preparation of deglycosylated ginsenosides	[153]
	Urea	ChCl: U	1:2		
	Ethylene glycol	ChCl: EG	1:2		
	1,4-Butanediol	ChCl: B	1:2		
	Glycerol	ChCl: G	1:1 & 1:2		
	Citric acid	ChCl: Ca	1:1		
	D-Glucitol	ChCl: Dg	1:1		
	Malic acid	ChCl: Ma	1:1		
	Xylitol	ChCl: X	1:2		
	Glucose	ChCl: Glu	5:2		
	Malic acid: Xylitol	ChCl: Ma: X	1:1:1		
	Urea: Glycerol	ChCl: U: G	1:1:1		
Betaine	Xylitol	BET: X	1:2 & 1:1 & 2:1		
	Glycerol	BET: G	1:1 & 1:2		
	Citric acid	BET: Ca	1:1		
	Ethylene glycol	BET: EG	1:2		
	Urea	BET: U	1:2		
	Malic acid	BET: Ma	1:1		
	Glucose	BET: Glu	5:2		
	Malic acid: Glucose	BET: Ma: Glu	1:1:1		
	Lysine	BET: Lys	1:1	▪ Novel BET-amino acid-based natural deep eutectic solvents for enhancing the enzymatic hydrolysis of corncob ▪ Potential solvent for biomass pretreatment	[154]
	Arginine	BET: Arg	1:1		
	Histidine	BET: His	1:1		
	Sorbitol	BET: S	1.25:1.2	▪ Optimization of BET-sorbitol natural deep eutectic solvent-based ultrasound-assisted extraction and pancreatic lipase inhibitory activity of chlorogenic acid and caffeine content from robust a green coffee beans ▪ BET-sorbitol NADES-UAE method provides an excellent extraction efficiency	[155]
	lactic acid	BET: La	1:2	▪ Application of BET-based natural deep eutectic solvents (NaDES), such as BET/lactic acid and BET/glycerol, in non-productive palm trunks and bioethanol production through efficient lignin degradation	[156]
	Glycerol	BET: G	1:2		
	Formic acid	BET: FA	1:2	▪ The removal of lignin and hemicellulose by pre-treatment of wheat straw with the KOH/BE: NE system increased enzymatic accessibility and improved enzymatic hydrolysis efficiency.	[157]
	Citric acid: water	BET: CA: W	1:2:3		
	Malic acid: water	BET: Ma: W	1:2:2		
	Polyethylene glycol 400: water	BET: PEG: W	1:2:4		
	Propylene glycol	BET: PG	1:2		
	Sorbitol: water	BET: SO: W	1:2:3		
	Methylurea: water	BET: ME: W	1:2:1		
	Acetamide: water	BET: AC: W	1:2:1		
	N-(2-hydroxyethyl) Ethylenediamine: water	BET: NE: W	1:2:4		

**Table 7:** The Key Points of Different Research Studies Considered BET-Based DESs in Pharmaceutical Applications.

**DES Components**		**Molar Ratio**	**Key Finding/Applications**	**References**
**Component 1**	**Component 2**			
BET	Benzoic	1:2	▪ Solubilisation of aromatic amino acids with low water solubility by this medium. ▪ Investigating the solubility of some *L*-amino acids was in this DES.	[164]
	2-Hydroxybenzoic (salicylic)			
	4-Chlorobenzoic			
	2-Chlorobenzoic			
	3-Chlorobenzoic			
	2-Furoic			
	Phenylacetic			
	*D*-(+)-Mandelic			
	Glycolic			
	Oxalic			
	Citric			
	Urea	1:1 1:1.5 1:2 1:2.5	▪ Highlighting the importance of BET and its role in protecting proteins from denaturation caused by urea.	[163]
	Glycerol	1:2	▪ Extraction of chlorogenic acid from *Lonicera japonica* Thunb. Using deep eutectic solvents: Process optimization and mechanism exploration by the combination of experiment and molecular simulation. ▪ BET/glycerol: superior extraction performance	[165]
	1,3-Propanediol	1:2		
	Ethylene Glycol	1:2		
	Lactic acid	1:1		
	Urea	1:2	▪ BET plays an important role in the pharmaceutical and healthcare industries, particularly in DES systems that enhance the dehydration of gabapentin	[167]
	Ascorbic acid: water	1:3:8 1:1:2 5:1:8	▪ Effectiveness of the therapeutic Deep Eutectic System (THEDES) based on *L*-ascorbic acid (AA) and BET, in the solubility and dermal delivery of AA and topical formulations to reduce signs of aging and even out skin tone	[115]

**Table 8:** The Key Points of Different Research Studies Considered BET-based DESs in Food Science and Preservation.

**DES Components**		**Molar Ratio**	**Key Finding/Applications**	**References**
**Component 1**	**Component 2**			
BET	Xylitol	3:2	▪ Extraction of oleanolic acid from grape waste using low-toxicity solvents DES (BET/xylitol) with high efficiency and milder conditions than conventional methods, for application in the food industry	[172]
	Glucose	5:2	▪ DESs improve antioxidant activity by up to 3 times by increasing the extraction and stability of plant compounds and are used in the food industry as promising active ingredients.	[173]
	Malic acid	1:1		
	Malic acid: proline	1:1:1		
	Malic acid: glucose	1:1:1		
	glycerol	1:2		
	Polyethylene glycol	1:3		
	ethylene glycol	1:3		
	Sorbitol: water	1:1:3		
	Glycerol	1:2	▪ DES-based packaging (CG and BG) showed a better effect in preserving the quality of cherry tomatoes than conventional samples (PE and Gel).	[174]
	Glycerol	1:4:40% water	▪ A green method for extracting beneficial phenolic compounds from grape skins demonstrated significant antibacterial activity in the BET/lactic acid DES extract.	[175]
	Lactic acid	1:4:40% water		
	Ascorbic acid	2:1:40% water		

## Data Availability

No new data were created or analyzed in this study. Data sharing is not applicable to this article.
